# BRD7 deficiency leads to the development of obesity and hyperglycemia

**DOI:** 10.1038/s41598-019-41713-0

**Published:** 2019-03-29

**Authors:** Junsik M. Lee, Yoo Kim, Mario Andrés Salazar Hernández, Youngah Han, Renyan Liu, Sang Won Park

**Affiliations:** Division of Endocrinology, Boston Children’s Hospital, Harvard Medical School, Boston, MA 02115 USA

## Abstract

Obesity is a debilitating disease that has become a global epidemic. Although progress is being made, the underlying molecular mechanism by which obesity develops still remains elusive. Recently, we reported that the expression levels of bromodomain-containing protein 7 (BRD7) are significantly reduced in the liver of obese mice. However, it is not clear whether decreased levels of hepatic BRD7 are directly associated with the development of obesity and disturbance in glucose homeostasis. Here, using heterozygous BRD7 knockout and liver-specific BRD7 knockout mouse models, we report that reduced BRD7 levels lead to increased weight gain with little effect on glucose metabolism. On the other hand, upregulating BRD7 in the liver starting at an early age protects mice from gaining excessive weight and developing glucose intolerance and insulin resistance when challenged with a high-fat diet.

## Introduction

Obesity is a global public health problem and a major risk factor behind several metabolic disorders such as type 2 diabetes and cardiovascular disease^1–3^. Despite the severity of health problems derived from obesity, understanding of the molecular mechanisms that lead to excessive weight gain and its associated pathologies is still not complete, and an effective method to treat obesity has not been established yet.

Bromodomain-containing proteins (BRDs) are evolutionarily conserved protein modules that recognize acetyl-lysine motifs^4^. BRD7 is a member of the BRD family with a molecular weight of about 75 kDa. It is ubiquitously expressed in most organs including the liver, brain, heart, colon, lung, and skin^5^, but the exact function of BRD7 is still not fully understood. It has been previously reported that BRD7 acts as a tumor suppressor by interacting with p53 and BRCA1^6–8^. More recently, it was discovered that BRD7 recognizes an acetylated lysine in vitamin D receptor (VDR) and directs its association to polybromo-associated BAF (PBAF) chromatin remodeling complex, while BRD9 promotes BAF-VDR association^9^. VDR shuttles between BRD7 and BRD9 in β cells and inhibition of BRD9 promotes PBAF-VDR association, which in turn acts to reverse β cell dysfunction and reduce blood glucose levels in diabetic mice.

In our previous work, we have shown that BRD7 is found in both the nucleus and cytoplasm. BRD7 interacts with the regulatory subunits of phosphatidylinositol 3-kinase (PI3K), p85α and p85β, and shuttles p85 to the nucleus^10,11^. This interaction between BRD7 and p85 leads to increased translocation of a transcription factor called the spliced form of X-box binding protein 1 (XBP1s) to the nucleus. The nuclear translocation process of XBP1s by BRD7 is further augmented by insulin stimulation^11^.

BRD7 levels are decreased in the liver of obese and type 2 diabetic mouse models^11^. Acute upregulation of BRD7 levels in the liver of *ob*/*ob* and diet-induced obese mice by the use of adenovirus-mediated gene transfer system leads to a decrease in blood glucose levels and improved glucose tolerance^11^. In addition, it was shown that BRD7 increases phosphorylation of glycogen synthase kinase 3β (GSK3β) and ribosomal protein S6 kinase (S6K)^12^. This phosphorylation is not observed in the absence of the mTORC1 signaling cascade. The increased phosphorylation of GSK3β and S6K was found to be robust even in the absence of AKT, while phosphorylation of eukaryotic translation initiation factor 4E-binding protein 1 (4E-BP1) and its effect on downstream eukaryotic translation initiation factor 4E (eIF4E) are not affected in the absence of AKT^12^.

While previous studies showed that BRD7 plays a role in insulin signaling and glucose homeostasis, the exact function of BRD7 in the development of obesity was unclear. In this report, we utilized knockout and transgenic mouse models to investigate how BRD7 affects the development of obesity and glucose homeostasis.

## Materials and Methods

### Animal lines

The whole body heterozygous BRD7 knockout mouse line (BRD7^+/−^) was obtained from the European Conditional Mouse Mutagenesis Program (EUCOMM)^13^. BRD7^+/−^ carries a cassette containing β‐galactosidase (LacZ) and neomycin‐resistance gene flanked by FRT sites. The cassette induces a frameshift and renders the *BRD7* gene non-functional. Liver-specific BRD7 knockout (LBKO) mice were generated by first crossing BRD7^+/−^ mice with a mouse line carrying Flp recombinase to restore the frameshift introduced by the LacZ-neomycin-resistance gene cassette. BRD7^+/−^ also carries LoxP sites flanking exon 5. Introduction of Cre recombinase by breeding offspring with a mouse line carrying Cre recombinase under a modified human albumin promoter produced LBKO mice (Supplementary Fig. S1)^12^. BRD7 transgenic (BRD7 Tg^+/−^) mice were generated by inserting a STOP sequence flanked by LoxP sites followed by BRD7 cDNA sequence under the CMV promoter at the ROSA 26 locus (Supplementary Fig. S1). Introduction of Cre deletes the STOP sequence, resulting in expression of BRD7. All mice were on a mixed 129/SvJ and C57BL/6J background. Genotypes were determined via polymerase chain reaction (PCR) using DNA extracted from tail biopsies collected at 3 weeks of age. Mice were fed either high-fat diet (HFD, 45% kcal from fat, Research Diets Inc., catalog number D12451) or normal chow diet (NCD, 14% kcal from fat, LabDiet, catalog number 5P76) and raised in a temperature and humidity controlled environment under a 12-hour light and dark cycle. All experiments were performed during the light cycle. All experiments were approved by Boston Children’s Hospital, Institutional Animal Care and Use Committee (IACUC) and performed in accordance with relevant guidelines and regulations.

### Glucose tolerance test (GTT)

Mice were fasted for 14 hours (7:00 p.m.–9:00 a.m.) and D-glucose solution was diluted in saline to a total volume of 100–150 μL. 1.5 g of D-glucose per kilogram body weight was administered by intraperitoneal injection. Blood glucose levels were measured from the tail using a commercial blood glucose meter (Bayer, model 9545 C) at 15, 30, 60, 90 and 120 minutes post-injection.

### Insulin tolerance test (ITT)

Mice were fasted for 6 hours (8:00 a.m.–2:00 p.m.) and recombinant human insulin from Eli Lilly was diluted in saline to a total volume of 100 μl. Diluted insulin was administered by intraperitoneal injection. Blood glucose levels were measured from the tail using a commercial blood glucose meter at 15, 30, 60, 90 and 120 minutes post-injection.

### Hepatic insulin infusion

Mice were fasted for 6 hours (8:00 a.m.–2:00 p.m.) and anesthetized with ketamine and xylazine. 100 μl saline or recombinant human insulin diluted in saline was infused into the liver through the portal vein. After 3 minutes of infusion, the liver was flash frozen in liquid nitrogen and stored at −80 °C.

### Adeno-associated virus (AAV) injection

Adeno-associated virus serotype 8 containing eGFP (AAV-GFP) or iCre (AAV-Cre) under the thyroxine-binding globulin promoter (Vector Biolabs, catalog number VB1743 and VB1724, respectively) was diluted in saline to a total volume of 100 μl per mouse. Mice were restrained in a restrainer and the AAV solution was injected through the tail vein. Mild pressure was applied at the spot of injection soon after injection to prevent the backflow of virus.

### Metabolic parameter measurement

Energy expenditure and respiratory exchange ratio were measured using a CLAMS open-circuit indirect calorimetry system (Columbus Instruments). Mice were housed individually in metabolic chambers. After 1 day of acclimatization, oxygen consumption (VO_2_, liters/hour), carbon dioxide production (VCO_2_, liters/hour), and movement of individual mice were measured every 18 minutes for 2 consecutive days. Respiratory exchange ratio (RER) and energy expenditure (EE, kcal/hour) were calculated as follows: RER = VCO_2_/VO_2_. EE = 3.815 × VO_2_ + 1.232 × VCO_2_. Movement was recorded by infrared light sources and optical sensors. The physical activity of each mouse was calculated by the total number of beam breaks.

### Body composition analysis

Body composition analysis was performed under isoflurane anesthesia using the PIXImus mouse densitometer (GE Lunar Corporation). No experiments were performed in the three days prior to analysis.

### Tissue lysis and western blotting

Liver tissues were homogenized with TissueLyser II (QIAGEN) in 1 ml of tissue lysis buffer composed of 25 mM Tris-HCl (pH 7.4), 10 mM Na_3_VO_4_, 100 mM NaF, 50 mM Na_2_P_2_O_7_, 10 mM EGTA, 10 mM EDTA, 1% NP-40, 2 mM PMSF, protease inhibitor cocktails (Roche, catalog number 11836170001) and phosphatase inhibitor cocktails (Roche, catalog number 04906837001). The homogenized samples were gently rotated on a tube rotator for 1 hour at 4 °C, followed by centrifugation at 16,100 × *g* for 1 hour at 4 °C. The lipid layer was removed using a cotton swab and supernatant was collected. Protein concentrations were measured using a protein assay kit (Bio-Rad Laboratories, catalog number 5000112) according to the manufacturer’s protocol. Normalized protein lysates were denatured in 1× Laemmli loading buffer by boiling at 100 °C for 5 minutes. The samples were loaded and resolved in an SDS-PAGE gel and then transferred onto polyvinylidene fluoride (PVDF) membrane at 4 °C. The membrane was blocked in Tris-buffered saline (TBS, pH 7.4) with 10% blocking reagent (Roche, catalog number 11921681001) for 1 hour at room temperature (RT). The membrane was incubated with primary antibody in TBS-Tween20 (TBS-T, pH 7.4) with 5% blocking reagent at 4 °C overnight. After incubation, the membrane was washed 3 times for 20 minutes with TBS-T at RT, followed by incubation with secondary antibody in TBS-T with 5% blocking reagent at RT for 1 hour. The membrane was washed 3 times for 20 minutes with TBS-T and then developed using a chemiluminescence assay system, and bands were visualized on autoradiography film. Primary antibodies to AKT, pAKT^Thr308^, pAKT^Ser473^, and HRP-linked secondary antibodies were purchased from Cell Signaling Technology. Antibody to tubulin was purchased from Cell Signaling Technology and Santa Cruz Biotechnology. BRD7 antibody was produced from Covance.

### Quantitative polymerase chain reaction (qPCR)

Total RNA was extracted from tissue samples using QIAzol Lysis Reagent (QIAGEN, catalog number 79306) according to the manufacturer’s protocol. cDNA was synthesized from 1 μg of RNA using the iScript cDNA Synthesis kit (Bio-Rad Laboratories, catalog number 1708890) according to the manufacturer’s protocol. After a 20x dilution in water, qPCR was performed with PowerUp SYBR Green Master Mix (Applied Biosystems, catalog number A25778) using the QuantStudio 6 Flex Real-Time PCR system (Applied Biosystems). The primer sequences are as follows:

*18s* F: 5′ AGTCCCTGCCCTTTGTACACA 3′

*18s* R: 5′ CGATCCGAGGGCCTCACTA 3′

*Brd7* F: 5′ GAGGCTGAGGTGTTCCAGAG 3′

*Brd7* R: 5′ TCACCTGGAGGCACTTGCTG 3′

*Pparγ* F: 5′ AGGCCGAGAAGGAGAAGCTGTTG 3′

*Pparγ* R: 5′ TGGCCACCTCTTTGCTCTGCTC 3′

*Fasn* F: 5′ GGAGGTGGTGATAGTCGGTAT 3′

*Fasn* R: 5′ TGGGTAATCCATAGAGCCCAG 3′

*Dgat2* F: 5′ TTCCTGGCATAAGGCCCTATT 3′

*Dgat2* R: 5′ AGTCTATGGTGTCTCGGTTGAC 3′

*Srebf1* F: 5′ GCGGTTGGCACAGAGCTT 3′

*Srebf1* R: 5′ GGACTTGCTCCTGCCATCAG 3′.

### Liver triglyceride content analysis

25 mg of liver tissue was homogenized with a bench-top homogenizer from Polytron (PT2100) in 2 mL of 50 mM sodium chloride (NaCl) solution in a round bottom glass tube for 30 seconds. 900 μl of homogenized sample was transferred to a capped glass tube. 5 mL of a mixed solution of molecular grade chloroform/methanol (2:1 vol/vol) was added to the homogenized sample and vortexed 3 times for 15 seconds. The samples were centrifuged at 500 × *g* for 10 minutes at 4 °C and the upper aqueous layer was discarded. 1.5 mL of methanol was added to the samples. A mixed solution of chloroform/methanol (2:1 vol/vol) was carefully added to each sample to a volume of 6 mL. Samples were then vortexed and centrifuged at 500 × *g* for 10 minutes at 4 °C. 75 μl of the lipid extract upper layer was transferred into a new glass tube. 18.75 μl of 10% Triton X-100 in acetone was added and the tubes were left to dry in a fume hood overnight. The dried pellet was dissolved in 400 μl of triglyceride reagent (Thermo Fisher, catalog number TR22421) and incubated at 37 °C for 20 minutes. After briefly vortexing, the absorbance at 650 nm was measured and subtracted from the absorbance at 500 nm and triglyceride content determined by standard curve comparison. To determine final triglyceride concentration in the liver, the triglyceride content calculated from the standard curve was first divided by 75 to account for the volume (μl) taken after the second centrifugation. This was multiplied by 6,000, which is the total volume (μl) of the chloroform/methanol solution. This number was divided by 0.45 to account for the initial volume (900 μl) that was used from 2 ml of homogenized sample.

### Plasma triglyceride concentration measurement

Blood samples were collected from the tail vein or through retro-orbital bleeding. Samples were centrifuged at 2,000 × *g* for 20 minutes at 4 °C and the supernatant was collected. 6 μl of plasma was added to 150 μl of triglyceride reagent (Thermo Fisher, catalog number TR22421) in a 96-well plate and incubated at 37 °C for 20 minutes. The absorbance at 650 nm was measured and subtracted from the absorbance at 500 nm and triglyceride content determined by standard curve comparison.

### Plasma insulin concentration measurement

Blood samples were collected from the tail vein or through retro-orbital bleeding. Samples were then centrifuged at 2,000 × *g* for 20 minutes at 4 °C and the supernatant was collected. Insulin concentration was measured using a mouse insulin ELISA kit (Crystal Chem, catalog number 90080) according to the manufacturer’s protocol.

### Quantification and statistical analysis

Immunoblots were quantified using the ImageJ software gel analyzer. Data were analyzed by Student’s t-test or two-way ANOVA. For data on body weight, GTT and ITT, two-way repeated measure ANOVA was used to examine the group differences at each time point. The Sidak’s method was used for the multiple comparison between groups. Data were shown as the mean ± SEM. Significance was presented at *p < 0.05, **p < 0.01 or ***p < 0.001.

## Results

### Reduced BRD7 levels lead to increased body weight

**Figure 1:**
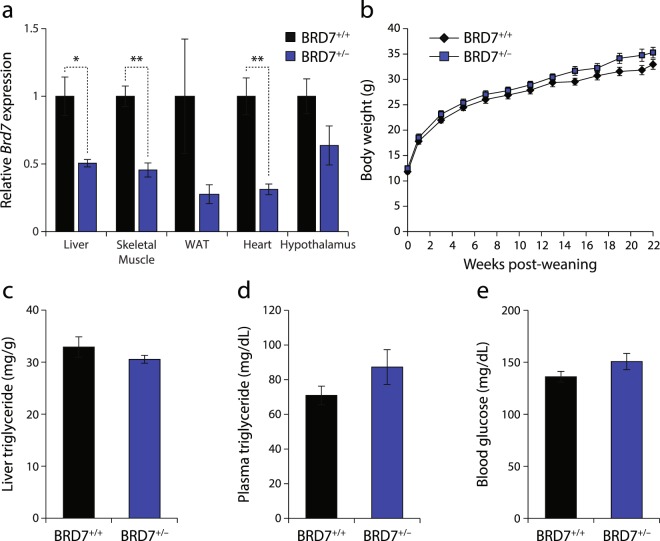
Reduced BRD7 levels lead to increased body weight. (**a**) Relative mRNA levels of *Brd7* normalized to *18 s* in the liver, skeletal muscle, white adipose tissue (WAT), heart, and hypothalamus. Mice were fasted for 6 hours. (**b**) Body weights of male BRD7^+/+^ and BRD7^+/−^ mice over 22 weeks after weaning (n = 8/group). (**c**) Liver triglyceride contents. Mice were fasted for 6 hours. (**d**) Plasma triglyceride levels. Mice were fasted for 6 hours. (**e**) Blood glucose levels at the 6-hour fasted state at week 5 post-weaning. Error bars are represented as mean ± SEM., *P* values were determined by Student’s *t*-test. (*p < 0.05, **p < 0.01, ***p < 0.001).

We have previously reported that homozygous knockout of BRD7 leads to embryonic lethality prior to embryonic day 16.5^13^. To understand the effect of BRD7 deficiency, we used heterozygous BRD7 knockout mice (BRD7^+/−^). The expression of *Brd7* mRNA was decreased in the BRD7^+/−^ mice by 49%, 54%, 72%, 69%, and 36% in the liver, skeletal muscle, white adipose tissue, heart, and hypothalamus, respectively, compared to their wild-type littermate controls (BRD7^+/+^) (Fig. 1a). Body weights and food intake were measured on a weekly basis for 22 weeks. BRD7^+/−^ mice displayed an increase in body weight compared to BRD7^+/+^ mice, but this increase did not reach statistical significance when analyzed by two-way ANOVA (Fig. 1b). There was no significant effect on food intake or body composition (data not shown). Liver triglyceride levels at the 6-hour fasted state were slightly reduced in BRD7^+/−^ mice (Fig. 1c), plasma triglyceride levels were increased (Fig. 1d), and blood glucose levels were elevated (Fig. 1e), but they did not reach statistical significance.

### The effect of reduced BRD7 levels on glucose homeostasis

**Figure 2:**
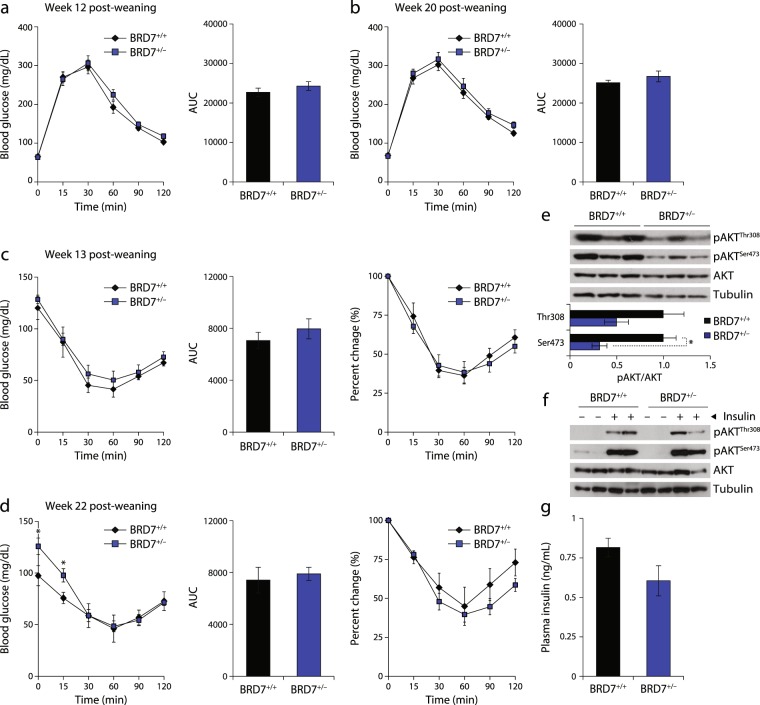
Reduced BRD7 levels have no effect on glucose homeostasis. (**a**) Glucose tolerance test (GTT) at week 12 post-weaning (left) (n = 9/group). Area under curve (AUC) of GTT (right). (**b**) GTT at week 20 post-weaning (left). Area under curve of GTT (right). (**c**) Insulin tolerance test (ITT) at week 13 (left). Area under curve of ITT (middle). Percent change of initial blood levels (right). (**d**) ITT at week 22 (left). Area under curve of ITT (middle). Percent change of initial blood glucose levels (right). (**e**) Immunoblots of pAKT^Thr308^ and pAKT^Ser473^ in the liver of BRD7^+/+^ and BRD7^+/−^ mice at 6 hours of fasting (top). Quantification of the blots showing the normalized ratio between pAKT and AKT (bottom). (**f**) Immunoblots of pAKT^Thr308^ and pAKT^Ser473^ in the liver of control and BRD7^+/−^ mice after infusion of saline or insulin (0.75 IU/kg) at 22 weeks (top). Quantification of the blots showing the normalized ratio between pAKT and AKT in insulin-infused livers (bottom). (**g**) Plasma insulin concentrations of 8-week old BRD7^+/+^ and BRD7^+/−^ mice at the 6-hour fasted state. Error bars are represented as mean ± SEM., *P* values were determined by Student’s *t*-test. (*p < 0.05, **p < 0.01, ***p < 0.001). Significance was determined by two-way analysis of variance (ANOVA) with Bonferroni multiple-comparison analysis.

To investigate the effect of reduced BRD7 on glucose metabolism, we performed a glucose tolerance test (GTT) and an insulin tolerance test (ITT) every 4 weeks post-weaning. There was no change in the response to an intraperitoneal injection of glucose between BRD7^+/−^ and BRD7^+/+^ mice throughout the course of the experiment (Fig. 2a,b). There was a slight difference between BRD7^+/−^ and BRD7^+/+^ mice in response to insulin at week 13 post-weaning as shown by an ITT (Fig. 2c), but this difference did not reach statistical significance. At week 22 post-weaning, fasting glucose levels were significantly increased in BRD7^+/−^ mice, then decreased to levels comparable to that of BRD7^+/+^ mice upon injection of insulin (Fig. 2d). Phosphorylation levels of AKT at residues Thr308 (pAKT^Thr308^) and Ser473 (pAKT^Ser473^) were determined by western blot. Both pAKT^Thr308^ and pAKT^Ser473^ were decreased in the liver of BRD7^+/−^ mice compared to that of BRD7^+/+^ mice at the 6-hour fasted state, but only the reduction in pAKT^Ser473^ was statistically significant (Fig. 2e). To further study insulin signaling, we infused insulin or saline as a control to the liver of BRD7^+/−^ and BRD7^+/+^ mice through the portal vein. There was no difference in pAKT^Thr308^ and pAKT^Ser473^ levels between BRD7^+/−^ and BRD7^+/+^ mice (Fig. 2f). The reduced AKT phosphorylation levels in BRD7^+/−^ mice at the 6-hour fasted state together with the fact BRD7^+/−^ mice still responded to insulin infusion led us to examine the plasma insulin levels. The insulin levels were decreased in BRD7^+/−^ mice compared to BRD7^+/+^ mice at the 6-hour fasted state, but the reduction did not reach statistical significance (Fig. 2g).

### HFD-challenged BRD7^+/−^ mice display increased weight gain

**Figure 3:**
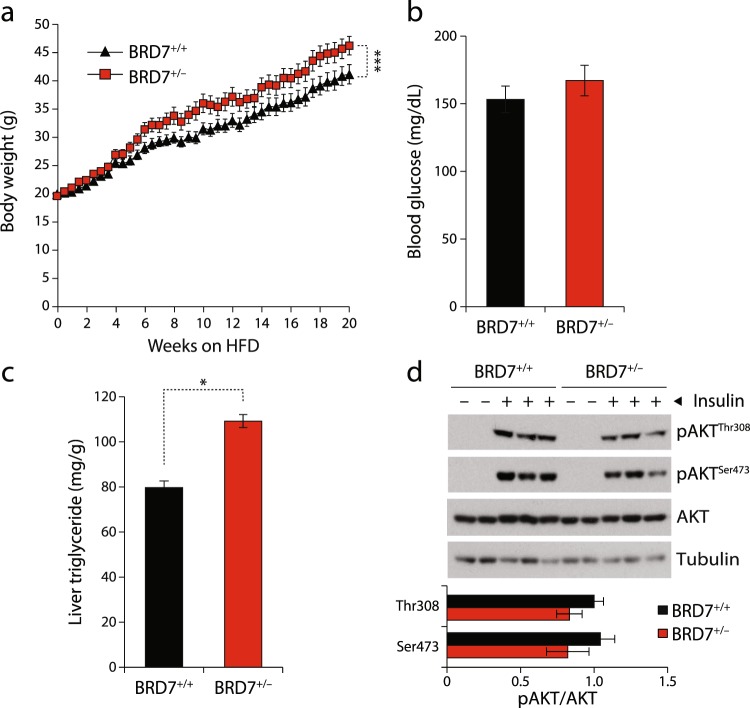
BRD7^+/−^ mice fed a HFD display increased body weights. (**a**) Body weights of male BRD7^+/+^ and BRD7^+/−^ mice over 20 weeks on a HFD (n = 10/each group). (**b**) Blood glucose levels after 6 hours of fasting on week 10 of HFD feeding. (**c**) Liver triglyceride contents. Mice were fasted for 6 hours. (**d**) Immunoblots of pAKT^Thr308^ and pAKT^Ser473^ in the liver of BRD7^+/+^ and BRD7^+/−^ mice after infusion of saline or insulin (1.5 IU/kg) after 22 weeks of HFD (top). Quantification of the blots showing normalized ratio between pAKT and AKT in insulin-infused livers (bottom). Error bars are represented as mean ± SEM., *P* values were determined by Student’s *t*-test. (*p < 0.05, **p < 0.01, ***p < 0.001). Significance was determined by two-way analysis of variance (ANOVA) with Bonferroni multiple-comparison analysis.

Given the increased body weights and higher blood glucose levels in BRD7^+/−^ mice, we asked whether feeding on a high-fat diet (HFD) combined with reduced whole body BRD7 levels would have a synergistic effect and result in further increased body weight gain and disturbed glucose homeostasis compared to HFD challenge alone. BRD7^+/+^ and BRD7^+/−^ mice were fed a HFD (45% kcal from fat) for 20 weeks and body weights and food intake were monitored on a weekly basis. BRD7^+/−^ mice started to display increased body weight at approximately week 5 on a HFD, and remained heavier throughout the course of the experiment (Fig. 3a). By week 20, BRD7^+/−^ mice gained 25% more body weight than BRD7^+/+^ mice. Blood glucose levels after 6 hours of fasting were higher in BRD7^+/−^ mice at week 10 (Fig. 3b), but the increase did not reach statistical significance. There was no significant difference in glucose tolerance and insulin sensitivity between BRD7^+/−^ mice and BRD7^+/+^ mice (data not shown). Meanwhile, BRD7^+/−^ mice showed a 37% increase in liver triglyceride content compared to the BRD7^+/+^ group (Fig. 3c). To understand whether hepatic insulin receptor signaling is altered with reduced BRD7 levels in HFD-challenged BRD7^+/−^ mice, we infused either insulin (1.5 IU/kg) or saline into the liver through the portal vein after 22 weeks of HFD. pAKT^Thr308^ and pAKT^Ser473^ levels were similar between the two groups after infusion (Fig. 3d).

### HFD-challenged liver-specific BRD7 knockout mice display increased weight gain

**Figure 4:**
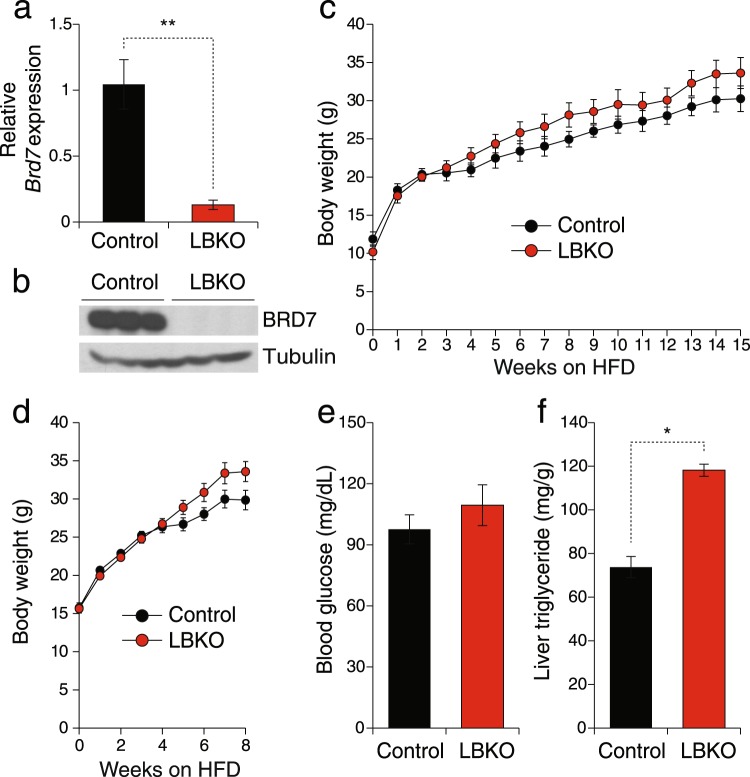
Liver-specific BRD7 knockout mice fed on a HFD display increased body weights. (**a**) Relative mRNA levels of *Brd7* normalized to *18 s* in the liver of LBKO and control mice at the 6-hour fasted state. (**b**) Immunoblot of BRD7 in the liver of LBKO and control mice. (**c**) Body weights of male control and LBKO mice over 15 weeks on a HFD (n = 7/group). (**d**) Body weights of control and LBKO mice over 8 weeks on a HFD (n = 7/group). (**e**) Blood glucose levels after 6 hours of fasting on week 8 of HFD feeding. (**f**) Liver triglyceride contents. Mice were fasted for 6 hours. Error bars are represented as mean ± SEM., *P* values were determined by Student’s *t*-test. (*p < 0.05, **p < 0.01, ***p < 0.001). Significance was determined by two-way analysis of variance (ANOVA) with Bonferroni multiple-comparison analysis.

Considering the fact that hepatic BRD7 levels are reduced in obese mice, we sought to investigate whether the lack of BRD7 in the liver has a major effect on body weight. First, we confirmed the successful generation of liver-specific BRD7 knockout (LBKO) mice by performing quantitative polymerase chain reaction (qPCR) (Fig. 4a) and western blotting (Fig. 4b). LBKO and corresponding control mice were placed on a HFD shortly after weaning and their body weights were monitored on a weekly basis for 15 weeks. LBKO mice gained more weight compared to control mice, but the difference did not reach statistical significance when analyzed by two-way ANOVA (Fig. 4c). Due to the fact that HFD itself decreases BRD7 levels^11^, the difference in BRD7 levels between control and LBKO mice after 15 weeks of HFD became insignificant (data not shown). Therefore, we repeated the experiment and monitored body weights of LBKO and control mice on a HFD for only 8 weeks. After 8 weeks, LBKO mice had higher body weights compared to control mice, but did not reach statistical significance (Fig. 4d). The 6-hour fasted blood glucose levels were higher in LBKO mice compared to control mice at week 8 on a HFD, but this difference did not reach statistical significance (Fig. 4e). We did not find any significant difference in glucose tolerance and insulin sensitivity between LBKO and control mice from GTT and ITT throughout the course of the experiment (data not shown). Liver triglyceride levels were significantly higher in LBKO mice compared to control mice (Fig. 4f).

### Sustained upregulation of BRD7 in the liver leads to long-term improvement in glucose homeostasis

**Figure 5:**
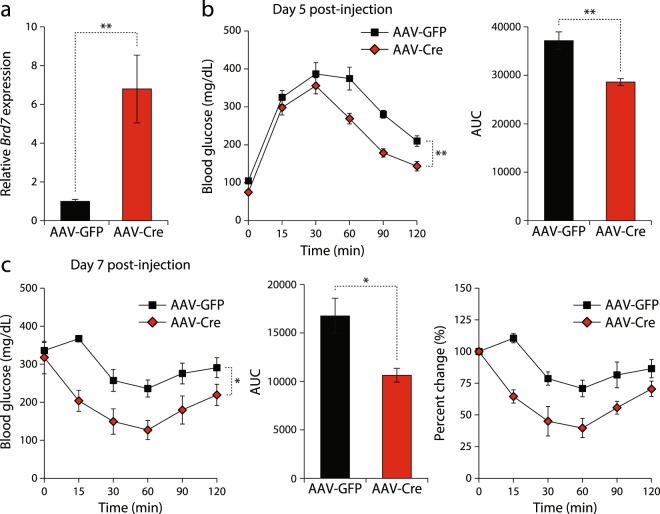
Upregulation of BRD7 in the liver at an early age improves glucose homeostasis. (**a**) Relative mRNA levels of *Brd7* normalized to *18 s* in the liver of HFD-fed male BRD7 Tg^+/−^ mice injected with AAV-GFP or AAV-Cre at week 11 post-injection (n = 5/group). (**b**) Glucose tolerance test on day 5 post-injection (left). Area under curve (right). (**c**) Insulin tolerance test on day 7 post-injection (left). Area under curve (middle). Percent change of basal levels (right). Error bars are represented as mean ± SEM., *P* values were determined by Student’s *t*-test. (*p < 0.05, **p < 0.01, ***p < 0.001). Significance was determined by two-way analysis of variance (ANOVA) with Bonferroni multiple-comparison analysis.

**Figure 6:**
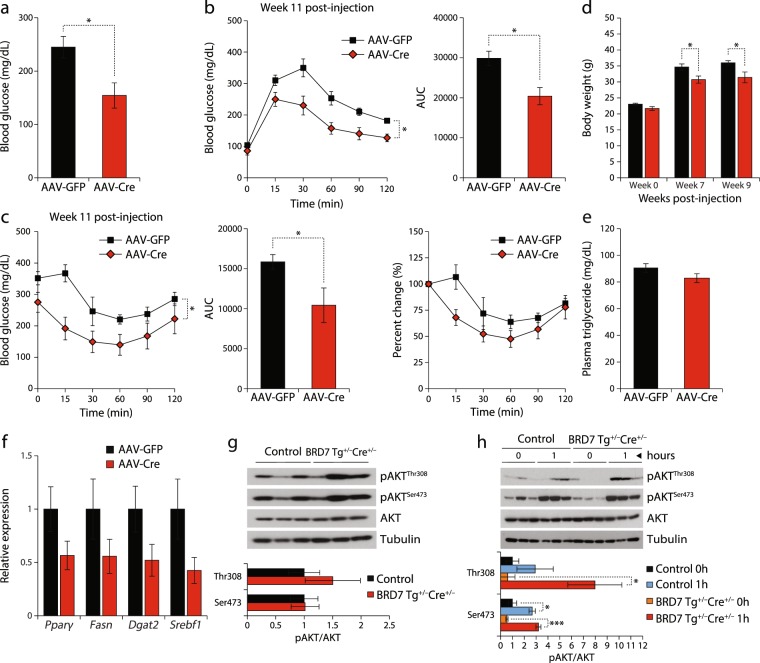
Sustained upregulation of BRD7 leads to long-term improvement in glucose homeostasis. (**a**) Blood glucose levels of HFD-fed male BRD7 Tg^+/−^ mice injected with AAV-GFP or AAV-Cre after 6 hours of fasting on day 12 post-injection (n = 5/group). (**b**) Glucose tolerance test on week 11 post-injection (left). Area under curve (right). (**c**) Insulin tolerance test on week 11 post-injection (left). Area under curve (middle). Percent change of initial blood glucose levels (right). (**d**) Body weights of AAV-GFP and AAV-Cre-injected mice at the time of injection and 7 and 9 weeks post-injection. (**e**) Plasma triglyceride levels 15 weeks post-injection. Mice were fasted for 6 hours. (**f**) Relative mRNA levels of *Pparγ*, *Fasn*, *Dgat2*, and *Srebf1* normalized to *18* *s* in the liver of HFD-fed male BRD7 Tg^+/−^ mice injected with AAV-GFP or AAV-Cre at week 11 post-injection. (**g**) Immunoblots of pAKT^Thr308^ and pAKT^Ser473^ in the liver of HFD-fed 9-week old male control and BRD7 Tg^+/−^Cre^+/−^ mice at 6 hours of fasting (top). Quantification of the blots showing normalized ratio between pAKT and AKT (bottom). (**h**) Immunoblots of pAKT^Thr308^ and pAKT^Ser473^ in the liver of HFD-fed 14-week old male control and BRD7 Tg^+/−^Cre^+/−^ mice at 24 hours of fasting and during 1 hour of refeeding following 24 hours of fasting (top). Quantification of the blots showing normalized ratio between pAKT and AKT in the liver of refed mice (bottom). Error bars are represented as mean ± SEM., *P* values were determined by Student’s *t*-test. (*p < 0.05, **p < 0.01, ***p < 0.001). Significance was determined by two-way analysis of variance (ANOVA) with Bonferroni multiple-comparison analysis.

Next, we sought to examine the effect of hepatic BRD7 upregulation on body weights and glucose metabolism. Understanding whether long-term overexpression of BRD7 in the liver is beneficial for glucose homeostasis would highlight its potential as a therapeutic target for the treatment of obesity and type 2 diabetes. For this purpose, BRD7 transgenic (BRD7 Tg^+/−^) male mice were placed on a HFD soon after weaning. In two different cohorts, we injected mice with AAV8-TBG-iCre (AAV-Cre) or AAV8-TBG-eGFP (AAV-GFP) at a dose of 1.4 × 10^11^ genome copies (gc) per mouse through the tail vein after 5 or 8 weeks of HFD feeding. The overexpression of Cre in BRD7 Tg^+/−^ mice deletes the STOP codon flanked by LoxP sites upstream of BRD7 cDNA and increases BRD7 expression levels (Supplementary Fig. S1). qPCR analysis showed that *Brd7* mRNA was increased by 6.8 fold in the liver after 22 weeks on a HFD (Fig. 5a). A GTT performed on day 5 post-injection showed significant improvement in glucose disposal rate in mice injected with AAV-Cre compared to the mice injected with AAV-GFP (Fig. 5b). An ITT performed on day 7 post-injection showed that insulin sensitivity was improved in the AAV-Cre-injected mice compared to AAV-GFP-injected mice (Fig. 5c). Moreover, AAV-Cre-injected mice displayed significantly lower 6-hour fasted blood glucose levels on day 12 post-injection (Fig. 6a). To assess whether the improvement in glucose metabolism was sustained long-term, we performed a GTT and an ITT on week 11 post-injection. Glucose disposal rate and insulin sensitivity remained significantly improved in AAV-Cre-injected BRD7 Tg^+/−^ mice (Fig. 6b,c).

Given that our BRD7^+/−^ and LBKO mice displayed increased weight gain and BRD7 upregulation significantly improved glucose homeostasis in AAV-Cre-injected BRD7 Tg^+/−^ mice, we sought to investigate whether the early upregulation of BRD7 could also protect against the development of obesity. For this purpose, we measured body weights of AAV-GFP and AAV-Cre-injected BRD7 Tg^+/−^ mice. Body weights of the AAV-Cre-injected mice were decreased by 11% at week 7 and 13% at week 9 compared to AAV-GFP-injected BRD7 Tg^+/−^ mice (Fig. 6d). There was no difference in food intake (data not shown).

Given the difference in body weight without a change in food intake, we questioned whether there was a difference in energy expenditure and energy source. To address this, we measured metabolic parameters in AAV-GFP and AAV-Cre-injected BRD7 Tg^+/−^ mice at week 15 post-injection. No differences were observed in energy expenditure (EE) and respiratory exchange ratio (RER) between the two groups in both the light and dark cycles (Supplementary Fig. S2). Plasma triglyceride levels were decreased in AAV-Cre-injected BRD7 Tg^+/−^ mice at the 6-hour fasted state, but it did not reach statistical significance (Fig. 6e). Considering the similarity in metabolic parameters between AAV-GFP and AAV-Cre-injected BRD7 Tg^+/−^ mice and a trend of decreased triglyceride levels in the plasma of AAV-Cre-injected BRD7 Tg^+/−^ mice, we questioned whether upregulation of BRD7 in the liver leads to any changes in white adipose tissue at the molecular level. We examined the expression of lipogenic genes in white adipose tissue. qPCR results showed reduced levels of *Pparγ*, *Fasn*, *Dgat2*, and *Srebf1* in AAV-Cre-injected BRD7 Tg^+/−^ mice compared to AAV-GFP-injected BRD7 Tg^+/−^ mice (Fig. 6f), but the reduction did not reach statistical significance.

To study the effect of upregulated BRD7 on insulin signaling, we bred BRD7 Tg^+/−^ mice with a mouse line that carries Cre recombinase under the albumin promoter (BRD7 Tg^+/−^Cre^+/−^). Mice were fed on a HFD for 12 to 16 weeks (three different cohorts). Phosphorylation levels of AKT were determined by performing western blot. pAKT^Thr308^ and pAKT^Ser473^ levels were increased in BRD7 Tg^+/−^Cre^+/−^ mice compared to control mice at the 6-hour fasted state, but the increase did not reach statistical significance (Fig. 6g). When mice were fasted for 24 hours and refed *ad libitum* for 1 hour, BRD7 Tg^+/−^Cre^+/−^ mice displayed higher pAKT^Thr308^ levels compared to control mice (Fig. 6h), but the difference did not reach statistical significance.

## Discussion

The prevalence of obesity is increasing worldwide. In 2016, the World Health Organization estimated that around 39% of the world’s adult population was overweight and of those, 33% was obese. This was a 25% increase since 2006. Recent studies reported that childhood and adolescent obesity places individuals at a higher risk for developing medical and psychological problems including type 2 diabetes, orthopedic issues, sleep apnea, asthma, depression, and cancer in adulthood^14–16^. In particular, the risk of developing type 2 diabetes is four times higher in children who are obese^17^. Therefore, it is important to prevent childhood obesity and the early development of its associated pathologies.

BRD7 has recently emerged as a player in glucose metabolism and the insulin signaling pathway^10–13^. BRD7 levels are significantly reduced in the liver of genetically obese *ob*/*ob* mice and HFD induced obese wild-type mice^11^. Therefore, we questioned whether decreased BRD7 levels play a role in the development of obesity and consequent type 2 diabetic features. In this report, we document that decreased BRD7 levels lead to the development of obesity. Our observations indicate that both heterozygous BRD7 knockout and liver-specific BRD7 knockout mice display increased body weight and liver triglyceride levels, which were further augmented when mice were challenged with HFD. Plasma insulin concentration was reduced in BRD7^+/−^ mice compared to BRD7^+/+^ mice. This may explain the decreased AKT phosphorylation levels in the liver of BRD7^+/−^ mice compared to control mice at the 6-hour fasted state. Reduced plasma insulin concentration was not observed in LBKO mice, suggesting that this reduction is facilitated by organs other than the liver. Wei Z, *et al*. recently reported a role of BRD7 in β cells of the pancreas^9^. They showed that the interaction of BRD7 with VDR ultimately leads to inhibition of BRD9 activity in β cells, which in turn reverses β cell dysfunction and reduces blood glucose levels in type 2 diabetic mouse models. Therefore, presumably β cell dysfunction due to decreased BRD7 in BRD7^+/−^ mice led to a reduction in plasma insulin concentration without a corresponding reduction of insulin levels in LBKO mice (Supplementary Fig. S3).

BRD7 deficiency exacerbated weight gain and BRD7 upregulation prevented the development of obesity. However, downregulation of BRD7 had little effect on glucose metabolism, while upregulation led to great improvement in glucose homeostasis during HFD-feeding. It is important to note that diet-induced obesity itself leads to markedly reduced hepatic BRD7 levels^11^ and it is not possible to establish a control group (HFD-induced obese mice with normal BRD7 expression levels) to study the effect of BRD7 deficiency in obesity. This makes it difficult to study the consequences of BRD7 downregulation once obesity has already developed. Nevertheless, our data suggest that reduced BRD7 levels play a role in the progression of obesity and hyperglycemia. Furthermore, here we clearly show that sustained overexpression of BRD7 levels in the liver of HFD-induced obese mice by AAV-mediated gene transfer system prevents excessive weight gain, significantly decreases blood glucose levels, and improves glucose metabolism. This implies that reduced levels of hepatic BRD7 per se may not directly lead to the development of type 2 diabetes, but preventing the decrease of BRD7 caused by obesity is enough to stop the progression of type 2 diabetes. To understand the mechanism behind the reduced weight gain in mice overexpressing hepatic BRD7, we monitored food intake and metabolic parameters. However, we observed no difference in food consumption, energy expenditure, or respiratory exchange ratio between AAV-GFP and AAV-Cre-injected BRD7 Tg^+/−^ mice. At the molecular level, the decrease in lipogenic genes in white adipose tissue of AAV-Cre-injected BRD7 Tg^+/−^ mice suggests inter-organ effects of hepatic BRD7. It is possible that the observed phenotypes are a consequence of this cross-talk between organs. However, what drives metabolic signals from the liver to other organs and how exactly hepatic BRD7 contributes to inter-organ metabolic signaling in response to HFD feeding warrant further investigation.

In conclusion, we have found that BRD7 upregulation protects against the development of obesity and also restores impaired glucose homeostasis once obesity has been established. Our work highlights BRD7 as a potential therapeutic target for the prevention of obesity and treatment for type 2 diabetes.

## Supplementary information

Supplementary figure
