# Supplementary material for: BRD7 deficiency leads to the development of obesity and hyperglycemia
Source: Sci Rep. 2019 Mar 29;9:5327. doi: 10.1038/s41598-019-41713-0 (PMC6441051; doi:10.1038/s41598-019-41713-0)
Supplement: Supplementary file 1 — Supplementary figure [file 41598_2019_41713_MOESM1_ESM.pdf]

BRD7 deficiency leads to the development of obesity and hyperglycemia.

Junsik M. Lee, Yoo Kim, Mario Andrés Salazar Hernández, Youngah Han, Renyan Liu and Sang Won Park*

Division of Endocrinology, Boston Children's Hospital, Harvard Medical School, Boston, MA 02115, USA

Supplementary Information

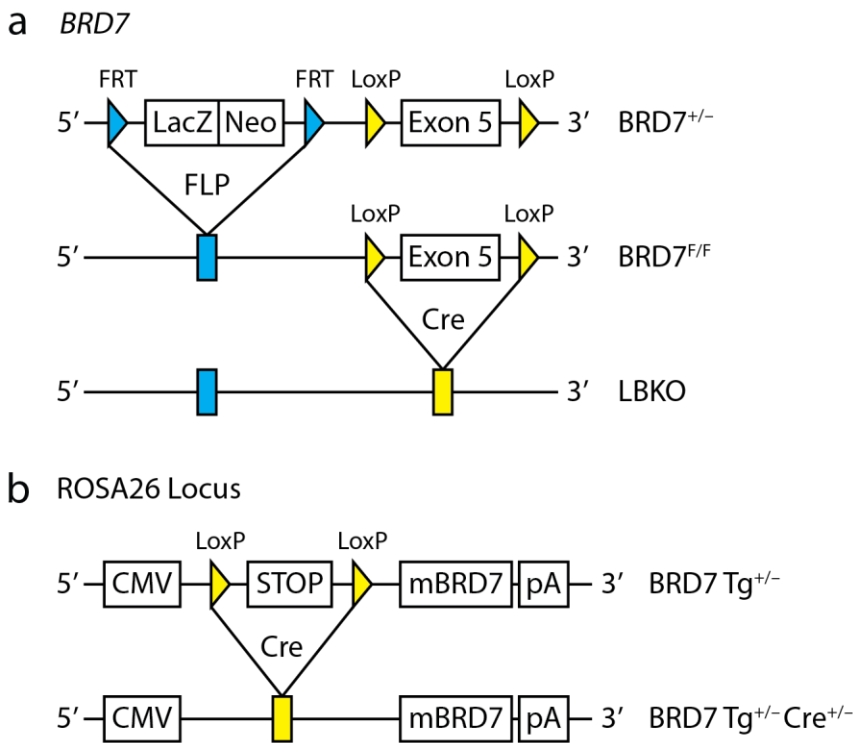

Figure S1. Schematic characterization of mouse lines. (**A**) Schematic of the modified *BRD7* allele in BRD7$^{+/-}$ mice (top) and LBKO mice following modification by FLP and Cre recombinase (bottom). (**B)** Schematic of the modified ROSA26 locus in BRD7 Tg$^{+/-}$ mice. pA indicates poly-A tail.

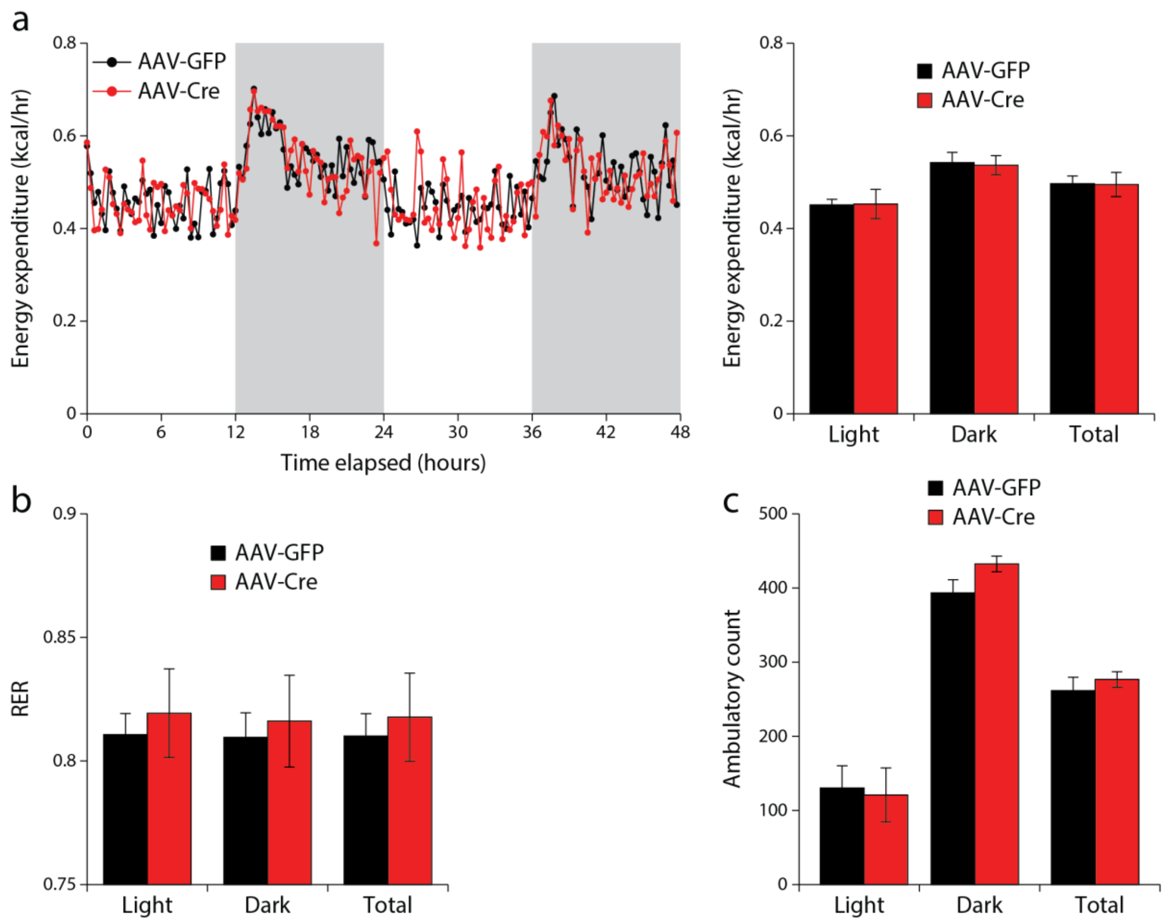

Figure S2. Metabolic parameters of AAV-GFP and AAV-Cre-injected BRD7 Tg$^{+/-}$ mice. (**A**) Energy expenditure of HFD-fed AAV-GFP and AAV-Cre-injected BRD7 Tg$^{+/-}$ mice at week 15 post-injection over the course of two light/dark cycles (left). Gray shading indicates the dark cycle. Average energy expenditure (right). (**B**) Average respiratory exchange ratio over two light/dark cycles. (**C**) Ambulatory count of each group. Bar graphs represent average over two light/dark cycles. Error bars are represented as mean ± SEM.

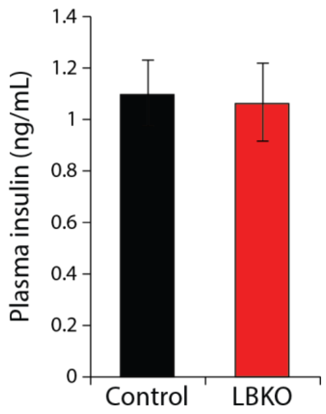

Figure S3. Plasma insulin levels of LBKO mice. Plasma insulin levels of 8-week old LBKO mice fed on a NCD (n=3). Mice were fasted for 6 hours. Error bars are represented as mean ± SEM.
